# Glucose fluctuations in association with oxidative stress among children with type 1 diabetes mellitus: comparison of different phases

**DOI:** 10.1186/1687-9856-2015-S1-P22

**Published:** 2015-04-28

**Authors:** Xi Meng, Chunxiu Gong, Bingyan Cao, Xiaoxia Peng

**Affiliations:** 1 Department of Endocrinology, Genetics and Metabolism, Beijing Children’s Hospital, Capital Medical University, Beijing, China; 2 Ministry of Education National Key Discipline of Pediatrics, Beijing Children’s Hospital, Capital Medical University, Beijing, China; 3 Center of clinical epidemiology and evidenced based medicine, Beijing Children’s Hospital, Capital Medical University, Beijing, China

**Keywords:** glucose fluctuations, oxidative stress, children, type 1 diabetes mellitus

## Objective

To examine the association of glucose fluctuation and oxidative stress in children with type 1 diabetes mellitus (T1DM) across different phases.

## Methods

Children treated for T1DM at Beijing Children’s hospital from 2010 to 2013 were enrolled and divided EQUALLY into three study groups including newly diagnosed children (Group A, acute metabolic disturbance phase), Group B (honeymoon phase), and Group C (long-standing phase). Healthy control children were matched to the T1DM patients by age and sex. The 24-hour urinary free 8-iso-prostaglandin F2α to creatinine (8-isoPGF2α/Cr) ratio indicated oxidative stress. Glucose fluctuation parameters (GFPs) included mean blood glucose levels (MBG), standard deviation of daily blood glucose levels (SDBG), mean amplitude of glucose excursions (MAGE), and incremental area under the curve for postprandial glucose (IAUC). GFPs and 8-isoPGF2α/Cr levels in the study groups were compared and the association of GFPs and 8-isoPGF2α/Cr across groups was assessed.

## Results

In each study group, 8-isoPGF2α/Cr and all GFPs in children with T1DM were significantly higher than those in normal controls. 8-isoPGF2α/Cr was significantly correlated with all GFPs in all three T1DM groups. Multiple linear regression analysis showed a stronger association with 8-isoPGF2α/Cr for MAGE than for HbA1c in both the acute metabolic disturbance and long-standing phases of T1DM.

## Conclusion

Glucose fluctuations were positively associated with oxidative stress inpatients in different phases of T1DM. Glucose fluctuations may have a stronger effect than sustained chronic hyperglycemia on triggering oxidative stress, but coexisting high levels of blood glucose are required.

